# Transferability of correlative and process‐based species distribution models revisited: A response to Booth

**DOI:** 10.1002/ece3.8081

**Published:** 2021-09-06

**Authors:** Steven I. Higgins, Matthew J. Larcombe, Nicholas J. Beeton, Timo Conradi

**Affiliations:** ^1^ Plant Ecology University of Bayreuth Bayreuth Germany; ^2^ Department of Botany University of Otago Dunedin New Zealand; ^3^ CSIRO Battery Point Tas. Australia; ^4^ School of Biological Sciences University of Tasmania Hobart Tas. Australia

**Keywords:** BIOCLIM, ecological niche model, invasive species, MaxEnt, mechanistic models, model transferability, TTR‐SDM

## Abstract

Here, we respond to Booth's criticism of our paper, “Predictive ability of a process‐based versus a correlative species distribution model.” Booth argues that our usage of the MaxEnt model was flawed and that the conclusions of our paper are by implication flawed. We respond by clarifying that the error Booth implies we made was not made in our analysis, and we repeat statements from the original manuscript which anticipated such criticisms. In addition, we illustrate that using BIOCLIM variables in a MaxEnt analysis as recommended by Booth does not change the conclusions of the original analysis. That is, high performance in the training data domain did not equate to reliable predictions in novel data domains, and the process model transferred into novel data domains better than the correlative model did. We conclude by discussing a hidden implication of our study, namely, that process‐based SDMs negate the need for BIOCLIM‐type variables and therefore reframe the variable selection problem in species distribution modeling.

## Graphical abstract

In response to a letter by Booth, we illustrate that using BIOCLIM variables in a MaxEnt analysis as recommended by Booth does not change the conclusions we drew in the original analysis. That is, high performance in the training data range did not equate to reliable predictions in novel data domains, and the process model we used transferred into novel data domains better than the correlative model did.

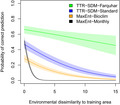

## 1 INTRODUCTION

We welcome the critique of our recent contribution to Ecology and Evolution (Higgins et al., 2020) by Dr Booth (Booth, 2021), and in this response, we take the opportunity to show that his concerns are unfounded and miss the point of Higgins et al. (2020). We begin by responding directly to the claims made in Booth's letter (Booth, 2021). The overarching argument is that it is possible to get better performance out of a correlative model than we did in Higgins et al. (2020) if you use different environmental variables. Booth suggests that using monthly data in MaxEnt is inappropriate because months are not comparable between hemispheres (January in the north is very different to January in the south), and BIOCLIM variables overcome this. Unfortunately, Booth is not precisely clear about what aspect of our analysis, which accounted for such seasonal differences (see below), is at fault, and he does not reveal how one objectively and a priori defines more appropriate variables. But he is clear that in his assessment our usage use of what he terms “inappropriate variables” is severe enough to cast doubt on the validity of our conclusions that process‐based models may have higher transferability than correlative species distribution models; specifically, Booth states that “These conclusions may be true, but readers cannot be sure as there is a major problem with the variables selected for use.”

We anticipated this type of criticism in the original manuscript when we wrote in the discussion that “It is likely that different MaxEnt decisions (pseudo‐absence sampling, regularization coefficients, feature selection, and clamping) would change the results we present here.” and that “In this study, we provided both models with the same presence, pseudo‐absence, and environmental data and use the default settings of the models. This favors the TTR‐SDM since it has rather precise data requirements and this study met those requirements. MaxEnt was, in contrast, forced to do the best it could with environmental data tailored for the TTR‐SDM.” Booth's letter draws attention to the validity of these statements, by explicitly providing an example of how using different variables in a MaxEnt model for *Acacia saligna* might enhance how MaxEnt performs in a transferability test.

Booth's letter implies that we ignored the fact that winter and summer fall in different months in different hemispheres. Indeed, if one did not relabel the months into a consistent seasonal sequence, this would compromise the between‐hemisphere transferability of the MaxEnt models we fitted. We relabelled months so that month 1 was the month after the winter solstice to avoid this problem when fitting and projecting the models. We unfortunately did make the error of not using this relabelling scheme when creating the zone map, which was used to stratify the sampling of presence and pseudo‐absence data. However, this would have only influenced the weighting of the environmental conditions considered in the samples. And since all model variants used the same sample points, this issue is unlikely to explain the differences in model performance revealed in Higgins et al. (2020); this is confirmed by the reanalysis we provide here. A corrected version of this zone map is in Figure S1 (cf. figure S1 in Higgins et al., 2020).

This leaves us with the task of illustrating that our conclusions remain valid even when using what Booth considers to be appropriate variables in the MaxEnt model. We thus ran an expanded analysis that included a MaxEnt model fit that uses the 19 BIOCLIM variables provided by Hijmans et al. (2005). That is, this expanded analysis uses MaxEnt‐Monthly (MaxEnt using monthly environmental variables as in the original manuscript), MaxEnt‐BIOCLIM (MaxEnt using BIOCLIM environmental variables), TTR‐STD (Standard TTR model), and TTR‐FQR (Farquhar TTR model). In the original manuscript, we used the R command rgbif::occ_search (Chamberlain et al., 2019) to download records from GBIF to create a data set of occurrences outside of the training data domain (outside of Australia). This unfortunately does not preserve attribution information on the data sources. In this reanalysis, we therefore use the R command rgbif::occ_download (Chamberlain et al., 2019) as this allows full attribution. The different download procedures and dates produced different occurrence data in the non‐native range. It is now possible to evaluate the model for 157 species instead of for 46 species as in the original manuscript. These differences in the GBIF test data do not influence the qualitative ranking or interpretation of the models performance outside of the training domain. Apart from these details, the protocol is as described in the original manuscript. We repeated all previous model fits for this analysis and slight differences in the model fits are present due to the usage of the corrected zone map (Figure S1) for the stratified random sampling of the occurrence pseudo‐absence records (see Higgins et al., 2020).

We do not present all the statistics presented in the original manuscript here: Instead, we focus on the AUC values in the training and test domain and reproducing expanded versions of figures 5 and 6 from the original manuscript. The AUC values derived for models evaluated against the training data (models fitted using the native range in Australia) when using MaxEnt‐BIOCLIM were intermediate between MaxEnt‐Monthly and the two TTR variants (median AUC values are, respectively, 0.992, 0.988, 0.971, and 0.969 for MaxEnt‐Monthly, MaxEnt‐BIOCLIM, TTR‐STD, and TTR‐FQR). The AUC values derived for models evaluated against observations made outside of the training data domain (in the non‐native range, outside of Australia) when using BIOCLIM variables for MaxEnt models are also intermediate between MaxEnt using monthly variables and the two TTR variants (median AUC values are, respectively, 0.594, 0.628, 0.703, and 0.777 for MaxEnt‐Monthly, MaxEnt‐BIOCLIM, TTR‐STD, and TTR‐FQR). That is, in this case study the rank order of AUC values are reversed in the training domain relative to the test data domain, suggesting that good performance in the training domain does not transfer to good performance in an independent testing domain.

**FIGURE 1:**
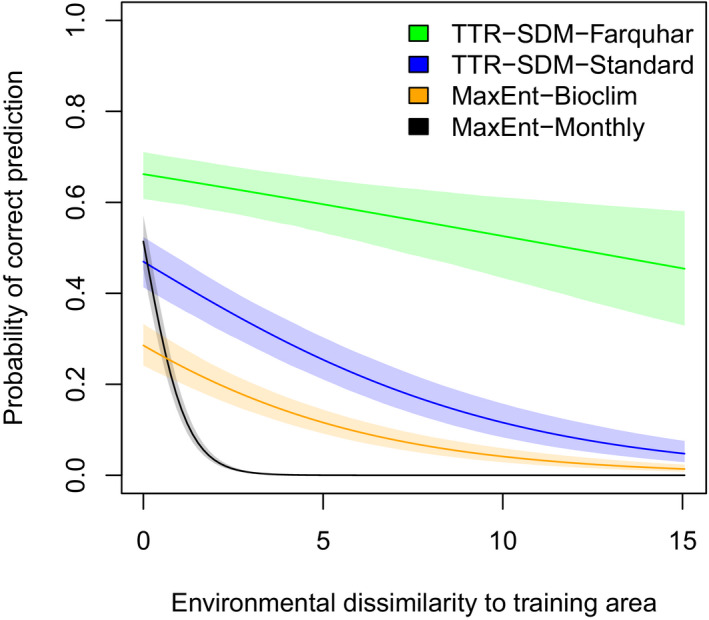
The probability of the species distribution models correctly predicting GBIF records of the 157 study species with sufficient data outside of Australia. The lines represent the fits of a Bayesian logistic regression model to an environmental dissimilarity score. The dissimilarity score indicates dissimilarity relative to the training arena. The shaded areas indicate the 95% credible intervals. This is a revised version of figure 6 from Higgins et al. (2020)

The probability of the models correctly predicting a GBIF observation outside of Australia (i.e., outside of the training domain) decreases with increasing environmental dissimilarity from the training data, where environmental dissimilarity is calculated using the monthly environmental data as in the original manuscript (Figure 1, cf. figure 6 in Higgins et al., 2020). The increased sample size (more species and more GBIF occurrence data) rescales the y axis of this graph relative to the original manuscript; that is, all models performed better when evaluated against this larger data set. However, the ranking remains the same. TTR‐FQR performs best and its predictive ability decays slower with increasing environmental dissimilarity than is the case for the other models. MaxEnt‐BIOCLIM is better than MaxEnt‐Monthly (as anticipated by Booth) but not as good as the two TTR‐SDM variants.

**FIGURE 2:**
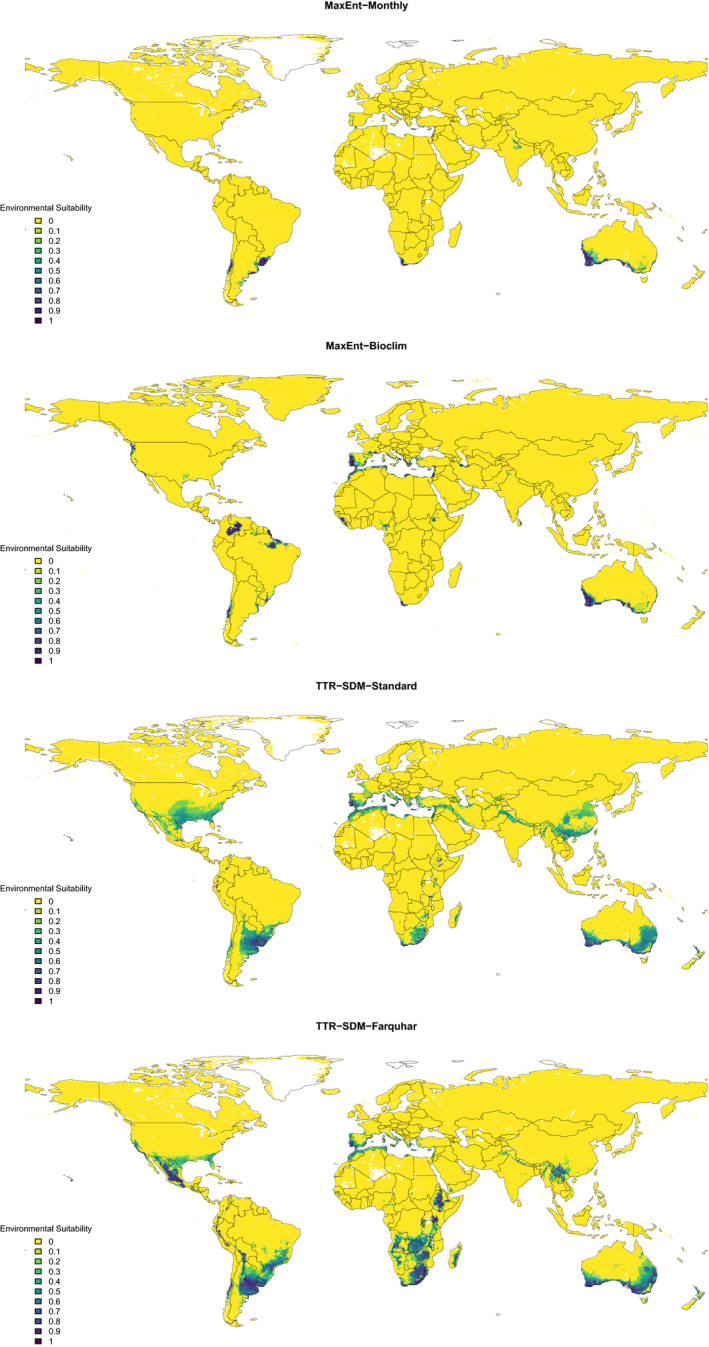
Global projected environmental suitability of *Acacia saligna* for four different species distribution models. The species distribution models were trained using data from Australia. The projections are made for 0.25 degree grid cells. This is a revised version of figure 5 from Higgins et al. (2020)

Figure 2 is a revision of our original figure 5 which used *Acacia saligna* to illustrate differences between the models' projections. This revised Figure should additionally be compared to Booth's figure 3. Booth's figure 3 suggests that he used only *Acacia saligna* records from western Australia to fit the model, whereas we used all Australian records in the original analysis. Furthermore, motivated by previous research (Thompson et al., 2011), Booth selected a subset of BIOCLIM variables. The model Booth fitted predicts *Acacia saligna* to be present in all Mediterranean‐type climate zones, but essentially nowhere else. Our MaxEnt‐BIOCLIM fit in turn uses all 19 BIOCLIM (Hijmans et al., 2005) variables, and although it less clearly identifies all Mediterranean‐type climate zones, it does identify some other regions such as Ethiopia where the species is observed in the GBIF records (see figure 5 in Higgins et al., 2020). The Booth analysis misses that parts of Ethiopia are suitable for *Acacia saligna*. The point is, however, not to get distracted into debating the best MaxEnt model for *Acacia saligna*: It is that a good fit in the training data domain is not necessarily a reliable indicator of predictive ability in a novel data domain. We should further not, in the context of our study, be distracted by discussions about MaxEnt variable selection procedures and ignore the message of our paper that process‐based species distribution models can be surprisingly transferable (Higgins et al., 2020). Indeed, process‐based species distribution models are maturing into alternatives to correlative models (Evans et al., 2016).

We also wish to draw attention to the fact that there is a significant conceptual difference in how environmental data is treated in process versus correlative species distribution models. In the TTR‐SDM, the model runs as a forward simulation, forced by monthly temperature, soil‐moisture, solar radiation, and soil nitrogen values. The model's equations are set up so that these environmental variables can colimit a plant's resource assimilation and growth. That is, the model simulates that a plant's monthly resource assimilation and growth are colimited by the temperature, soil–moisture, solar radiation, and soil nutrients that a plant is exposed to each month of a year. This means that the model can directly mimic the fact that soil–water limits growth in the Mediterranean summer, whereas solar radiation and temperature may colimit in the Mediterranean winter. This essentially means that process‐based models do not need BIOCLIM‐type variables, which attempt to develop proxies for colimitation processes such as the temperature in the driest quarter or rainfall in the warmest quarter. That is, process‐based models allow us to use our knowledge of plant physiology along with data on the fundamental environmental variables (light, water, temperature, and nutrients) that influence plant performance to make inferences about the potential ranges of plant species. Fortunately, there are now increasingly sophisticated numerical libraries available for simulating microclimatic variables needed for forcing process‐based species distribution models (Kearney et al., 2020). The challenge in process‐based species distribution modeling is therefore not defining and selecting the forcing variables, but rather defining how to represent the ecophysiology of colimitation processes in elegant and efficient ways. Using process models frees us from the impossible task of producing climatic indices that correlate with the outcome of time‐dependent colimitation dynamics.

Overall, the differences in how different SDMs use environmental data make model comparisons difficult and the increasing diversity of models mean that comprehensive comparisons will soon require prohibitive effort. Booth's critique of our attempt to benchmark a boutique method (TTR‐SDM) against a widely used method (MaxEnt) is a symptom of this problem. In Higgins et al. (2020), we suggested that data sets for benchmarking methods should be established allowing developers of new methods to compare their analyses to analyses conducted by developers of existing methods. A model for how this could work was demonstrated by Magarey et al. (2018) who asked experts in different SDM methods to analyze the same data in the context of a transferability test.

## CONFLICT OF INTEREST

None declared.

## AUTHOR CONTRIBUTION

**Steven I Higgins:** Conceptualization (lead); Formal analysis (lead); Writing‐original draft (lead). **Matthew Larcombe:** Conceptualization (equal); Writing‐review and editing (equal). **Nicholas Beeton:** Conceptualization (equal); Writing‐review and editing (equal). **Timo Conradi:** Conceptualization (equal); Writing‐review and editing (equal).

## Supporting information

Fig S1

## Data Availability

The data used in this study were downloaded from open‐access databases www.gbif.org (GBIF occurrence downloads https://doi.org/10.15468/dl.g3g2ed and https://doi.org/10.15468/dl.uua9kc) and www.avh.chah.org.au (AVH downloads https://doi.org/10.26197/5cf641a2df07c and https://doi.org/10.26197/5cf641c6aa20f).
