# Supplementary figures and images for: Transferability of correlative and process‐based species distribution models revisited: A response to Booth
Source: Ecol Evol. 2021 Sep 6;11(19):13613–7. doi: 10.1002/ece3.8081 (PMC8495818; doi:10.1002/ece3.8081)

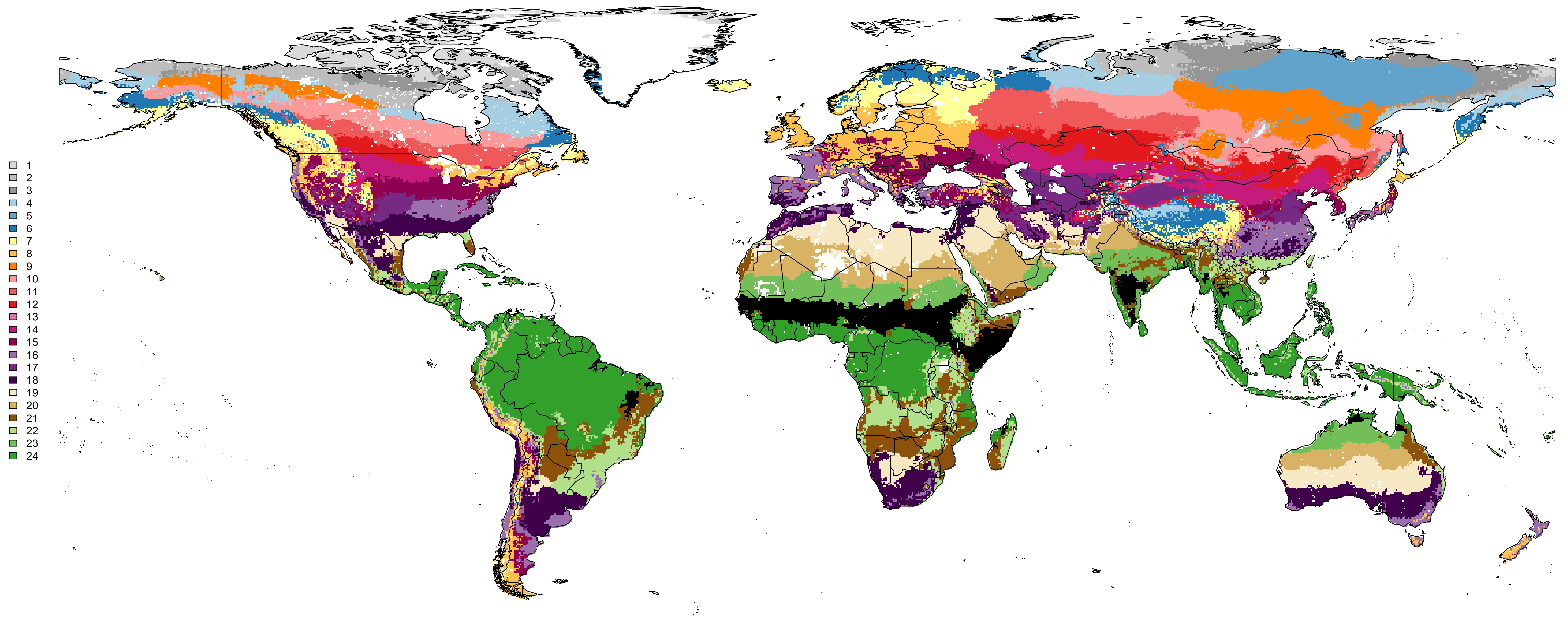

Supplement: Supplementary file 1 — Fig S1 [file ECE3-11-13613-s001.pdf]
